# Challenges and Opportunities for Reducing Low-Value Care; A Response to Recent Commentaries

**DOI:** 10.34172/ijhpm.2023.7954

**Published:** 2023-03-05

**Authors:** Simone A. Van Dulmen, Eva W. Verkerk, Karen Born, Reshma Gupta, Gert P. Westert, Rudolf B. Kool

**Affiliations:** ^1^Radboud Institute for Health Sciences, IQ healthcare, Radboud University Medical Center, Nijmegen, The Netherlands; ^2^Institute for Health Policy, Management & Evaluation, University of Toronto, Toronto, ON, Canada; ^3^University of California Health, Los Angeles, CA, USA

 Six commentaries have been published in reaction to our article “Key Factors That Promote Low-Value Care: Views From Experts From the United States, Canada, and the Netherlands.”^1^ In our original article, we have interviewed de-implementation experts in Canada, the United States and the Netherlands. We identified key national-level factors affecting the use of low-value care in those countries. The authors of the six commentaries have added valuable remarks to our key factors and mentioned several other factors that might play a role in stimulating low-value care. This is helpful in showing the complexity of reducing low-value care. There are recommendations to reduce low-value care across many countries, however these are challenging to implement. In this response, we highlight a few issues that, in our opinion, might help the international healthcare community in taking the next step in de-implementing low-value care.

## Organizational Culture and Habits

 Ingvarsson et al^2^ highlight that there should be more emphasis on strategies to target the organizational culture of ‘more is better.’ We agree that this is an extremely important factor which should be addressed on different levels. A key issue in changing organizational culture for effective de-implementation is a behavioral approach. Clinical behaviour of clinicians involves reflective and automatic processes. Reflective processes are reasoned behaviours based on knowledge about facts and values. Automatic processes are described as habits and routines and rely on heuristic decision-making.^3^ Habits and routines can be difficult to change, even when the clinician is aware that change is necessary. Habits and routines are embedded in established behavioural patterns and require a serious and structural effort to change.^4^ De-implementation strategies should therefore target these habits and routines in order to change organisational culture to address both reflective and automatic processes that drive clinician behaviour.

## Policy Strategies to Counter Uncertainty

 The role of uncertainty as an influencing element of physician behavior and patient preferences vis-à-vis low-value care is mentioned by van Bodegom-Vos et al.^5^ They suggest several strategies to reduce uncertainty, such as strategic reframing of non-medical approaches or offering a substitute to the low-value care practice. We absolutely agree with these suggestions. We want to add to this that system-focused strategies such as standardization, automation and forcing functions are generally more effective than person-focused strategies such as education.^6^ Therefore, for sustainable de-implementation, we need also policy changes with influence on a system level. Targeting an entire country or region is extremely efficient, as opposed to more local and temporary strategies such as education or audit and feedback. These macro level changes might make it easier for clinicians and patients to resist the meso and micro level factors that drive low-value care and choose the high-value care option. Strategies such as stopping reimbursement or strong recommendations or statements from clinician associations can provide the backup that a clinician needs in order to communicate confidently to a patient that a care practice is of low-value. Policy-level or macro strategies to support shared decision-making could be programs that aim to train clinicians, educate patients, or develop shared-decision making tools. Patients can also directly be targeted with this message by mass communication. For example, patients and the public can be directly targeted with media content and information related to low-value care.

## Structured Process of De-Implementation

 The authors of all commentaries agree that reducing low-value care by changing clinician and patient behaviour is an undeniable challenge, as many factors are expected to be interdependent and this affects the most effective strategy to reduce low-value care.^2,7^ As low-value care is also often driven by multiple factors, it is not likely that addressing only one factor with a strategy significantly reduces low-value care. Clinicians all over the world might be helped by a structured approach to change behaviour based on (de)implementation literature. The Choosing Wisely De-implementation Framework might be a helpful tool to choose, develop and evaluate tailormade interventions in a systematic and rigorous manner.^8^ The framework proposes a process to develop theory-informed interventions based on the following questions: Who needs to do what differently?; what barriers and enablers need to be addressed?; what intervention components could overcome the barrier and enhance the enablers?; and how will we measure behaviour change? The framework provides a guidance on five phases: (1) identification of potential areas of low-value healthcare; (2) identification of local priorities for de-implementation of low-value care; (3), identification of barriers and enablers and potential interventions to overcome these; (4) rigorous evaluations of the de-implementation program; and (5) spread of the program. The framework provides guidance on choosing de-implementation interventions for key actors (eg, patients, healthcare providers, managers and policy makers) to change their behaviours and/or decisions whilst working in complex and often chaotic healthcare environments.

## Evaluation and Research

 We strongly agree with the conclusion present in all commentaries that we need advanced research on how to avoid and reduce low-value care. Zadro et al^9^ emphasize the need for a review on effective interventions. Although there are several reviews on the effect of de-implementation interventions,^10-13^ none of these reviews provide a clear guidance on the type of intervention for a certain low-value care practice or context. We need especially more evidence on how to develop and evaluate interventions, and moreover preserve results and spread effective de-implementation interventions. A key and also a challenging step is the selection of the right interventions for the identified barriers and enablers. Clinicians might be helped by frameworks such as the Behavioral Change Wheel.^14^ Recently, the available evidence was collected and used to generate an online tool for linking behavior change techniques and mechanisms of action.^15^ For the successful spread of effective interventions, recently a framework was published to support the scaling of de-implementation strategies.^16^

## Conclusion

 Low-value is a complex phenomenon, present across healthcare systems globally. In order to reduce low-value care we need strategies on different levels that target the barriers so clinicians and patients will change their behaviour. Policy changes that can reduce uncertainty in clinicians and patients regarding low-value care are promising, but need to be used with caution. Clinicians should be supported to lead de-implementation initiatives. More knowledge and experience with de-implementation can spur the high-value care movement and inspire more clinicians to change their practice. That could help us making a significant next step forwards to a sustainable high-value healthcare system.

## Ethical issues

 Not applicable.

## Competing interests

 Authors declare that they have no competing interests.

## Authors’ contributions

 SAvD, EWV, and RBK wrote the first draft of the manuscript. RG, KB, and GW contributed expertise in low-value care and healthcare overuse. All authors read and approved the final manuscript.
